# 3-(2-Nitro­phen­oxy)phthalonitrile

**DOI:** 10.1107/S1600536807067797

**Published:** 2008-01-04

**Authors:** Xian-Fu Zhang, Dandan Jia, Aijun Song, Qiang Liu

**Affiliations:** a Department of Chemistry, Hebei Normal University of Science and Technology, Qinhuangdao, Hebei Province 066004, People’s Republic of China; b Department of Chemistry, Tsinghua University, Beijing 100084, People’s Republic of China

## Abstract

In the title compound, C_14_H_7_N_3_O_3_, the dihedral angle between the two arene units is 62.57 (12)°.

## Related literature

For related literature, see: Atalay *et al.* (2003 ▶, 2004 ▶); Cave *et al.* (1986 ▶); Köysal *et al.* (2004 ▶); Leznoff & Lever (1989–1996 ▶); McKeown (1998 ▶); Ocak Ískeleli (2007 ▶); Ocak *et al.* (2003 ▶), Sharman & van Lier(2003 ▶).
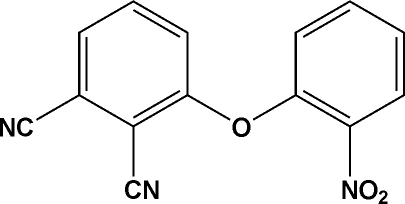


## Experimental

### 

#### Crystal data

C_14_H_7_N_3_O_3_
*M* *_r_* = 265.23
Monoclinic, 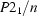

*a* = 8.0814 (17) Å
*b* = 7.9899 (12) Å
*c* = 19.068 (3) Å
β = 95.944 (15)°
*V* = 1224.6 (4) Å^3^
*Z* = 4
Mo *K*α radiation
μ = 0.10 mm^−1^
*T* = 295 (2) K
0.4 × 0.4 × 0.1 mm

#### Data collection

Bruker *P*4 diffractometer
Absorption correction: none
3018 measured reflections
2155 independent reflections
1252 reflections with *I* > 2σ(*I*)
*R*_int_ = 0.092
3 standard reflections every 97 reflections intensity decay: none

#### Refinement

*R*[*F* ^2^ > 2σ(*F* ^2^)] = 0.067
*wR*(*F* ^2^) = 0.145
*S* = 1.03
2155 reflections
181 parameters
H-atom parameters constrained
Δρ_max_ = 0.20 e Å^−3^
Δρ_min_ = −0.27 e Å^−3^

### 

Data collection: *XSCANS* (Bruker, 1997 ▶); cell refinement: *XSCANS*; data reduction: *XSCANS*; program(s) used to solve structure: *SHELXTL* (Bruker, 1997 ▶); program(s) used to refine structure: *SHELXTL*; molecular graphics: *SHELXTL*; software used to prepare material for publication: *SHELXTL*.

## Supplementary Material

Crystal structure: contains datablocks I, global. DOI: 10.1107/S1600536807067797/gd2031sup1.cif

Structure factors: contains datablocks I. DOI: 10.1107/S1600536807067797/gd2031Isup2.hkl

Additional supplementary materials:  crystallographic information; 3D view; checkCIF report

## Figures and Tables

**Fig. 1.**
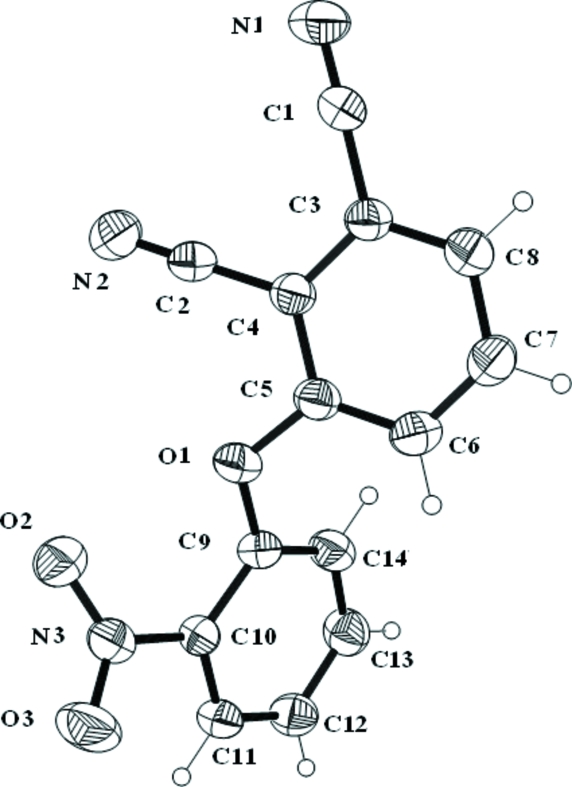
The molecular structure of C14H7N3O3 with 35% probability ellipsoids, showing the atom numbering scheme.

## supplementary crystallographic information

### Comment

Phthalonitriles are among the most important precursors of phthalocyanine materials (Leznoff, 1989–1996). Monophneoxyphthalonitriles have been used for preparing symmetrical phthalocyanines which have been applied in many areas, such as laser printing, photocopying, optical data storage, and catalysis (McKeown, 1998).

In the title compound, (I), (Fig. 1) the triple bond lengths between C and N, 1.136 (5) Å and 1.129 (5) Å, agree with literature values (Ocak *et al.*, 2003). The geometry around the O atoms is in good agreement with the literature (Atalay *et al.*, 2003, 2004; Köysal *et al.*, 2004). The dihedral angle between the two intramolecular arene moieties is 62.57 (12)°.

### Experimental

*o*-nitrophenol (1.39 g, 10.0 mmol) and 3-nitrophthalonitrile (1.73. g, 10.0 mmol) were dissolved in dry DMF (15 ml) with stirring under N_2_. Dry fine-powdered potassium carbonate (2.5 g, 18.1 mmol) was added over the course 1 h in equal portions every 10 min. The reaction mixture was stirred for 48 h at room temperature and poured into iced water (150 g). The product was filtered off and washed with(10% *w*/*w*) NaOH solution and water until the filtrate was neutral. Recrystallization from ethanol gave a white product (yield 1.72 g, 65%). Single crystals were obtained from absolute ethanol at room temperature *via* slow evaporation (m.p. 397–400 K). IR data (ν _max/cm^-1^): 3050(Ar—H), 1591(NO_2_), 2230(CN). NMR δ(H) 7.34–7.39(1*H*, m), 7.53–7.63(2*H*,m), 7.81–7.93(3*H*,m), 8.19–8.26(1*H*,m).

### Refinement

H atoms were included as riding atoms in geometrically idealized positions with C—H distances 0.93 Å and *U*_iso_(H) = 1.2*U*_eq_(C).

### Crystal data

**Table:**

C_14_H_7_N_3_O_3_	*F*_000_ = 544
*M**_r_* = 265.23	*D*_x_ = 1.439 Mg m^−^^3^
Monoclinic, *P*2_1_/*n*	Mo *K*α radiation λ = 0.71073 Å
Hall symbol: -P 2yn	Cell parameters from 51 reflections
*a* = 8.0814 (17) Å	θ = 5.0–12.5º
*b* = 7.9899 (12) Å	µ = 0.11 mm^−^^1^
*c* = 19.068 (3) Å	*T* = 295 (2) K
β = 95.944 (15)º	Plate, colorless
*V* = 1224.6 (4) Å^3^	0.4 × 0.4 × 0.1 mm
*Z* = 4	

### Data collection

**Table:**

Bruker P4 diffractometer	*R*_int_ = 0.092
Radiation source: fine-focus sealed tube	θ_max_ = 25.0º
Monochromator: graphite	θ_min_ = 2.2º
*T* = 295(2) K	*h* = −1→9
ω scans	*k* = −9→1
Absorption correction: none	*l* = −22→22
3018 measured reflections	3 standard reflections
2155 independent reflections	every 97 reflections
1252 reflections with *I* > 2σ(*I*)	intensity decay: none

### Refinement

**Table:**

Refinement on *F*^2^	Secondary atom site location: difference Fourier map
Least-squares matrix: full	Hydrogen site location: inferred from neighbouring sites
*R*[*F*^2^ > 2σ(*F*^2^)] = 0.067	H-atom parameters constrained
*wR*(*F*^2^) = 0.146	*w* = 1/[σ^2^(*F*_o_^2^) + (0.001*P*)^2^ + 1.2*P*] where *P* = (*F*_o_^2^ + 2*F*_c_^2^)/3
*S* = 1.03	(Δ/σ)_max_ < 0.001
2155 reflections	Δρ_max_ = 0.20 e Å^−^^3^
181 parameters	Δρ_min_ = −0.27 e Å^−^^3^
Primary atom site location: structure-invariant direct methods	Extinction correction: none

### Special details

**Table:**

Geometry. All e.s.d.'s (except the e.s.d. in the dihedral angle between two l.s. planes) are estimated using the full covariance matrix. The cell e.s.d.'s are taken into account individually in the estimation of e.s.d.'s in distances, angles and torsion angles; correlations between e.s.d.'s in cell parameters are only used when they are defined by crystal symmetry. An approximate (isotropic) treatment of cell e.s.d.'s is used for estimating e.s.d.'s involving l.s. planes.
Refinement. Refinement of *F*^2^ against ALL reflections. The weighted *R*-factor *wR* and goodness of fit *S* are based on *F*^2^, conventional *R*-factors *R* are based on *F*, with *F* set to zero for negative *F*^2^. The threshold expression of *F*^2^ > σ(*F*^2^) is used only for calculating *R*-factors(gt) *etc*. and is not relevant to the choice of reflections for refinement. *R*-factors based on *F*^2^ are statistically about twice as large as those based on *F*, and *R*- factors based on ALL data will be even larger.

### Fractional atomic coordinates and isotropic or equivalent isotropic displacement parameters (Å^2^)

**Table:**

	*x*	*y*	*z*	*U*_iso_*/*U*_eq_	
O1	0.4115 (3)	0.5913 (3)	0.63769 (12)	0.0683 (8)	
O2	0.5975 (4)	0.8586 (4)	0.66974 (15)	0.0806 (9)	
O3	0.7869 (4)	0.8009 (4)	0.75304 (16)	0.0932 (11)	
N1	0.0726 (6)	0.4464 (5)	0.36401 (19)	0.0967 (14)	
N2	0.1821 (5)	0.7972 (5)	0.50408 (18)	0.0822 (11)	
N3	0.6493 (4)	0.7811 (4)	0.72224 (17)	0.0609 (9)	
C1	0.1511 (5)	0.4108 (5)	0.4142 (2)	0.0651 (11)	
C2	0.2279 (5)	0.6648 (6)	0.51586 (18)	0.0566 (10)	
C3	0.2487 (5)	0.3691 (5)	0.47972 (18)	0.0534 (9)	
C4	0.2840 (4)	0.4955 (5)	0.53013 (17)	0.0492 (9)	
C5	0.3749 (4)	0.4568 (5)	0.59366 (18)	0.0524 (9)	
C6	0.4322 (5)	0.2954 (5)	0.6072 (2)	0.0623 (10)	
H6A	0.4952	0.2702	0.6495	0.075*	
C7	0.3950 (5)	0.1729 (5)	0.5576 (2)	0.0657 (11)	
H7A	0.4328	0.0644	0.5667	0.079*	
C8	0.3024 (5)	0.2082 (5)	0.49420 (19)	0.0620 (10)	
H8A	0.2765	0.1234	0.4615	0.074*	
C9	0.4213 (4)	0.5689 (5)	0.71071 (17)	0.0523 (9)	
C10	0.5397 (4)	0.6628 (4)	0.75215 (18)	0.0480 (9)	
C11	0.5535 (5)	0.6431 (5)	0.82497 (18)	0.0584 (10)	
H11A	0.6334	0.7036	0.8530	0.070*	
C12	0.4516 (5)	0.5362 (5)	0.85555 (19)	0.0603 (10)	
H12A	0.4628	0.5220	0.9042	0.072*	
C14	0.3162 (5)	0.4646 (5)	0.7422 (2)	0.0626 (11)	
H14A	0.2348	0.4043	0.7148	0.075*	
C13	0.3319 (5)	0.4496 (5)	0.8141 (2)	0.0643 (11)	
H13A	0.2599	0.3792	0.8352	0.077*	

### Atomic displacement parameters (Å^2^)

**Table:**

	*U*^11^	*U*^22^	*U*^33^	*U*^12^	*U*^13^	*U*^23^
O1	0.090 (2)	0.0637 (18)	0.0462 (14)	−0.0149 (15)	−0.0149 (13)	−0.0003 (13)
O2	0.090 (2)	0.082 (2)	0.0670 (18)	−0.0169 (17)	−0.0075 (16)	0.0199 (16)
O3	0.0672 (19)	0.119 (3)	0.088 (2)	−0.0375 (19)	−0.0167 (17)	0.0071 (19)
N1	0.128 (4)	0.087 (3)	0.065 (2)	0.014 (3)	−0.036 (2)	−0.007 (2)
N2	0.113 (3)	0.067 (3)	0.063 (2)	0.008 (2)	−0.005 (2)	0.0033 (19)
N3	0.064 (2)	0.062 (2)	0.0549 (19)	−0.0103 (18)	−0.0035 (17)	−0.0013 (17)
C1	0.082 (3)	0.056 (2)	0.054 (2)	−0.005 (2)	−0.009 (2)	−0.0072 (19)
C2	0.065 (3)	0.062 (3)	0.0408 (19)	−0.002 (2)	−0.0030 (18)	−0.0008 (19)
C3	0.060 (2)	0.056 (2)	0.0429 (19)	−0.005 (2)	−0.0001 (17)	0.0012 (18)
C4	0.049 (2)	0.053 (2)	0.0451 (19)	−0.0043 (18)	0.0015 (15)	−0.0005 (17)
C5	0.056 (2)	0.056 (2)	0.0438 (19)	−0.006 (2)	0.0004 (17)	−0.0018 (18)
C6	0.064 (2)	0.068 (3)	0.053 (2)	0.001 (2)	−0.0055 (19)	0.006 (2)
C7	0.080 (3)	0.057 (2)	0.059 (2)	0.007 (2)	0.002 (2)	0.006 (2)
C8	0.076 (3)	0.057 (3)	0.053 (2)	0.000 (2)	0.004 (2)	−0.0059 (19)
C9	0.056 (2)	0.053 (2)	0.045 (2)	0.0009 (19)	−0.0076 (17)	0.0009 (17)
C10	0.049 (2)	0.043 (2)	0.050 (2)	0.0003 (17)	−0.0030 (16)	−0.0021 (16)
C11	0.062 (2)	0.059 (2)	0.051 (2)	−0.002 (2)	−0.0103 (19)	−0.0059 (18)
C12	0.065 (3)	0.068 (3)	0.047 (2)	−0.001 (2)	0.0039 (19)	−0.0027 (19)
C14	0.059 (2)	0.065 (3)	0.061 (2)	−0.016 (2)	−0.007 (2)	0.001 (2)
C13	0.066 (3)	0.065 (3)	0.062 (2)	−0.011 (2)	0.010 (2)	0.002 (2)

### Geometric parameters (Å, °)

**Table:**

O1—C5	1.377 (4)	C6—H6A	0.9300
O1—C9	1.398 (4)	C7—C8	1.383 (5)
O2—N3	1.214 (4)	C7—H7A	0.9300
O3—N3	1.213 (4)	C8—H8A	0.9300
N1—C1	1.129 (5)	C9—C14	1.372 (5)
N2—C2	1.136 (5)	C9—C10	1.395 (5)
N3—C10	1.452 (5)	C10—C11	1.390 (5)
C1—C3	1.445 (5)	C11—C12	1.359 (5)
C2—C4	1.443 (6)	C11—H11A	0.9300
C3—C8	1.376 (5)	C12—C13	1.372 (5)
C3—C4	1.403 (5)	C12—H12A	0.9300
C4—C5	1.385 (5)	C14—C13	1.369 (5)
C5—C6	1.385 (5)	C14—H14A	0.9300
C6—C7	1.372 (5)	C13—H13A	0.9300
			
C5—O1—C9	119.6 (3)	C3—C8—C7	119.8 (3)
O3—N3—O2	123.6 (4)	C3—C8—H8A	120.1
O3—N3—C10	117.4 (3)	C7—C8—H8A	120.1
O2—N3—C10	119.0 (3)	C14—C9—C10	119.9 (3)
N1—C1—C3	178.2 (5)	C14—C9—O1	122.7 (3)
N2—C2—C4	179.1 (4)	C10—C9—O1	117.4 (3)
C8—C3—C4	119.8 (3)	C11—C10—C9	119.0 (4)
C8—C3—C1	121.4 (3)	C11—C10—N3	118.5 (3)
C4—C3—C1	118.7 (3)	C9—C10—N3	122.5 (3)
C5—C4—C3	119.3 (3)	C12—C11—C10	120.6 (3)
C5—C4—C2	120.1 (3)	C12—C11—H11A	119.7
C3—C4—C2	120.5 (3)	C10—C11—H11A	119.7
O1—C5—C4	114.8 (3)	C11—C12—C13	119.5 (4)
O1—C5—C6	124.5 (3)	C11—C12—H12A	120.3
C4—C5—C6	120.5 (3)	C13—C12—H12A	120.3
C7—C6—C5	119.4 (3)	C13—C14—C9	119.6 (4)
C7—C6—H6A	120.3	C13—C14—H14A	120.2
C5—C6—H6A	120.3	C9—C14—H14A	120.2
C6—C7—C8	121.1 (4)	C14—C13—C12	121.3 (4)
C6—C7—H7A	119.4	C14—C13—H13A	119.3
C8—C7—H7A	119.4	C12—C13—H13A	119.3
